# Olfactory Neuroblastomas: An Experience of 24 Years

**DOI:** 10.5402/2011/451086

**Published:** 2011-06-20

**Authors:** Deniz Tural, Ozcan Yildiz, Fatih Selcukbiricik, Mehmet Akif Ozturk, Yildiz Keles, Buge Oz, Omer Uzel, Gokhan Demir, Nil Molinas Mandel

**Affiliations:** ^1^Division of Medical Oncology, Department of Internal Medicine, Cerrahpasa Medical Faculty, Istanbul University, 34098 Istanbul, Turkey; ^2^Deparment of Radiation Oncology, Medical Park Hospital, Antalya, Turkey; ^3^Department of Pathology, Cerrahpasa Medical Faculty, Istanbul University, Istanbul, Turkey; ^4^Department of Radiation Oncology, Cerrahpasa Medical Faculty, Istanbul University, Istanbul, Turkey; ^5^Division of Medical Oncology, Department of Internal Medicine, Istanbul Bilim University, Istanbul, Turkey

## Abstract

*Objective*. The aim of this study was to evaluate clinicopathological findings and the efficacy of the treatment modalities used in patients with olfactory neuroblastomas. *Study Design*. Retrospective record review. *Setting*. Istanbul University, Cerrahpasa Medical Faculty, medical oncology outpatient clinic. *Subjects and Methods.* There were 3 stage A tumors, 5 stage B and 11 stage C according to the Kadish staging system. There were 5 grade I/II and 12 grade III/IV according to the Hyams' histopathologic system. Involvement to orbita was detected in eight patients at the time of diagnosis. *Results*. The median follow-up period was 23.7 months. The 5-year survival rate for the whole group was 26%. The stage A/B groups exhibited a better survival rate than the C group with 2-year survival rates being 25 versus 71% respectively (*P* = .008). The grade I/II groups exhibited a better survival rate than the grade III/IV groups with 2-year survival rates being 50 versus 16% respectively (*P* = .001). The group who had orbital involvement exhibited a poor survival rate than the group of patients who had no involvement of the orbital. *Conclusion*. In our study, tumor stage, histopathologic grading, involvement of the orbita, brain and bone marow metastases were the statistically significant prognostic factors.

## 1. lntroduction

Olfactory neuroblastoma (ONB) is a rare malignant tumor arising from the olfactory epithelium of the nasal cavity and the paranasal sinuses. Berger et al. first described this uncommon neoplasm in 1924 [1]. This tumor commonly invades paranasal sinuses and the adjacent structures. The anatomic origin in the superior nasal cavity leads to nonspecific symptoms that make early diagnosis difficult. Direct extension of the tumor into the anterior cranial fossa, either at presentation or at disease recurrence, is common. The most common symptoms at presentation were reported as nasal stuffiness and intermittent epistaxis. ONB is mostly a locally aggressive tumor that can spread to lymph nodes but metastatic involvement of bone, bone marrow, and other organs was also reported. The most commonly used staging system proposed by Kadish is currently based on computer tomography and magnetic resonance imaging. In stage A, the tumor is limited to the nasal cavity, in stage B the tumor extends to paranasal sinus, and in stage C the tumor is extending over the nasal cavity and sinus and/or metastasized distantly [2]. A histological grading system based on microscopic findings proposed by Hyams at 1988 showed a clear correlation of survival with histological differentiation of disease [3]. 

Because of the rarity of olfactory neuroblastoma there is no consensus on its management. However most authors believe that craniofacial resection followed by RT offers the gold standard treatment in this disease. Despite aggressive therapies, local, regional relapse, and distant metastasis occur frequently and often after extended periods of follow-up. Data for this case series were acquired retrospectively from the files of Division of Medical Oncology, Cerrahpasa Medical Faculty, Istanbul University. Additional information regarding the clinical course and outcome was collected from the patients' charts and phone calls to the patients, their relatives, and their general practitioners. In this study, we have reviewed 19 cases of olfactory neuroblastomas treated over 24-year period and analyzed the clinical features, treatment outcomes, and prognostic factors. 

## 2. Patients and Methods

The data of nineteen patients of olfactory neuroblastomas treated and followed up between 1986 and 2010 at Division of Medical Oncology, Cerrahpasa Medical Faculty, Istanbul University, were analyzed retrospectively. Patient characteristics, initial symptoms, tumor extent, histologic features, primary therapy, tumor recurrence, and treatments of recurrent disease were determined. Diagnosis of ONB was based on histopathologic features and radiologic localization of the tumor. Patients were staged according to the Kadish staging system. A single pathologist with expertise in head and neck neoplasms reviewed the histological specimens. We also classified our cases according to Hyams' histopathologic grading system. Local ethics committee's approval and patients' or their next of kin's informed consent were taken before the study. 

## 3. Statistical Analysis

Treatment response was assessed by clinical examinations and computed tomography (CT) or magnetic resonance imaging (MRI). Survival rates were calculated from diagnosis to the death of the patient using the Kaplan-Meier life table method. Curves were compared using the log-rank test. The analyses were performed using SPSS 15.0 (SPSS Inc. Chicago, IL, USA) software. 

## 4. Results

### 4.1. Patient Characteristics

19 patients (10 males/9 females) were included in the analysis. The madian age at diagnosis was 46 (range: 19 to 68 years). The peak incidence occurred at the fourth decade (5/19, 38%). Tumor staging was Kadish stage A in 3, stage B in 5, and stage C in 11 patients. There were 5 grade I/II and 12 grade III/IV according to the Hyams' histopathologic grading system. There was involvement to orbita in eight patients at the time of diagnosis. Four patients had metastasis of cervical lymph nodes, two brains, one bone marrow, one bone and lung metastases, and one bone, mediastinal lymph node, and liver metastases at the time of diagnosis. Tumor localization, clinical symptoms, and presentations at the time of diagnosis are listed in Table 1. Median time from onset of first symptoms until diagnosis was 8.8 months (range: 1.8–29 months). The common presenting symptoms were nasal obstruction (53%), epistaxis (31%), rhinorrhea, and facial pain (26%). Patient characteristics, treatment modalities, and outcome are summarized in Table 2. 

### 4.2. Treatment

Patient treatments consisted of surgical resection, radiation therapy, and chemotherapy (CTX) (Table 3). Initial treatments included surgery alone in three patients, radiotherapy (RT) with or without CTX in five, surgery plus postoperative RT in seven and multimodality therapy (surgery plus postoperative CTX plus postoperative RT) in three, and finally CTX alone in one. In 14 patients, craniofacial resection was the primary treatment modality. Four of these patients had also radical neck dissection due to cervical lymph nodes involvement. Nasal tumor resection in 2 patients, maxillectomy plus orbital exenteration in 1, and external ethmoidectomy (EE) in 1 were also used as surgical treatments. Of these 14 patients who underwent surgical tumor resection, 4 were treated with surgery alone, 5 with adjuvant radiation therapy after surgery, 2 with adjuvant CTX after surgery, and the remaining 3 with radiation therapy and CTX after surgery. Of the remaining 5 patients, four in advanced stage and one in early stage refused surgical treatment. Radiation therapy was administered to two of the patients, radiation therapy followed by CTX to the other two, and CTX alone to one patient. The average radiation dose was 57.9 Gy and the CTX was chosen from cisplatin-based regimens. As a part of the primary treatment modality, 12 patients underwent radiation therapy, so did the five relapsed patients. As a part of primary treatment modality 8 patients received CTX, so did the three relapsed patients. 

### 4.3. Survival

**Figure 1:**
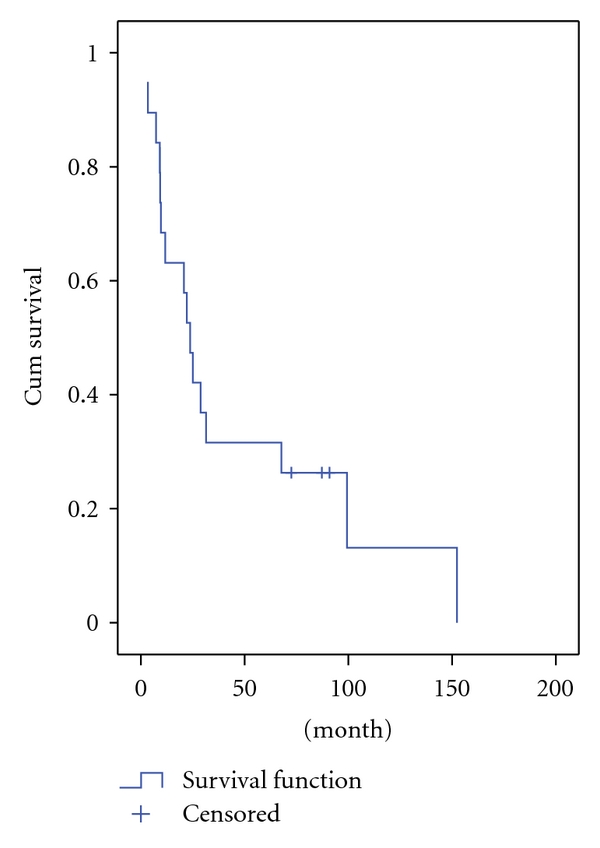
Overall survival in patients with olfactory neuroblastoma.

**Figure 2:**
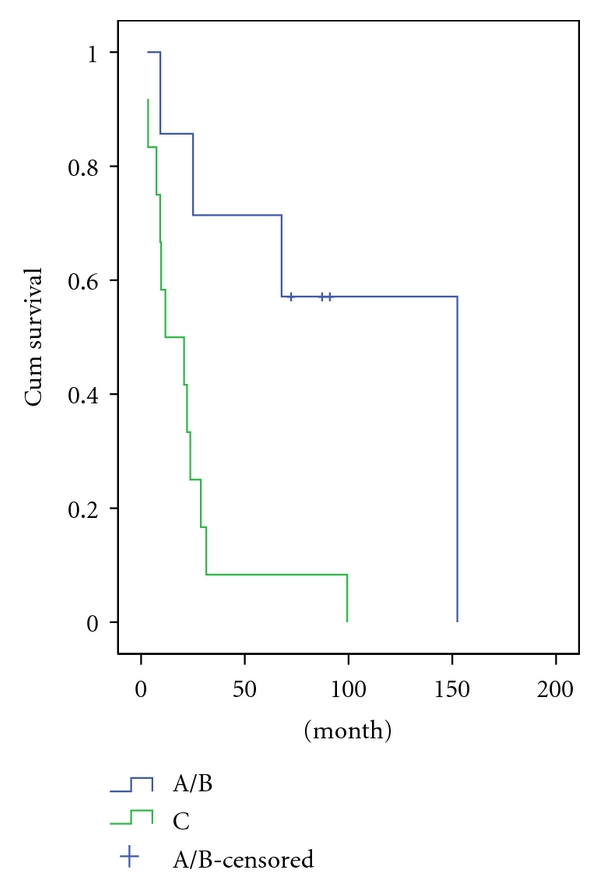
Survival in patients with olfactory neuroblastoma according to Kadish stage.

**Figure 3:**
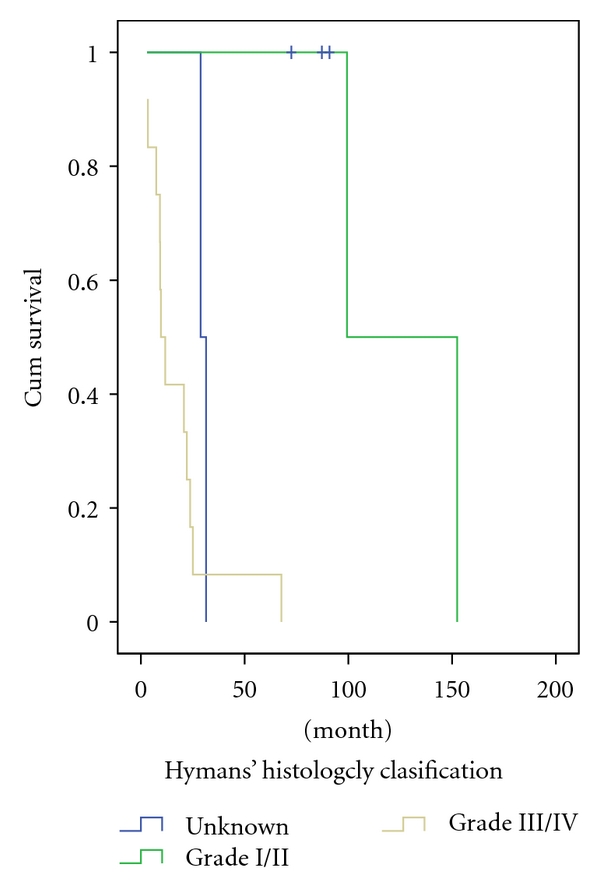
Survival in patients with olfactory neuroblastoma according to Hyams' histopathological classification.

**Figure 4:**
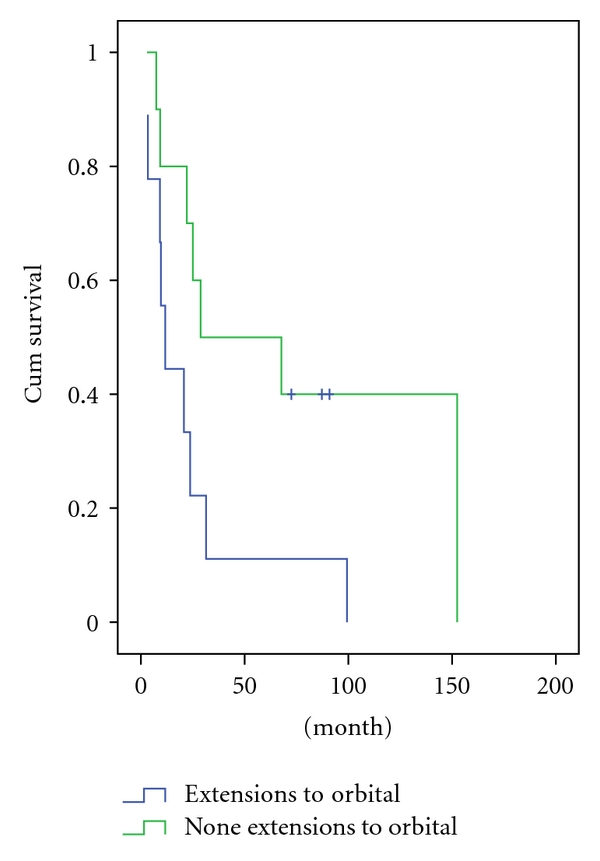
Survival in patients with olfactory neuroblastoma according to orbital involvement.

The 5-year survival rate of all patients was 26%. The mean survival time was 23 months (Figure 1). Fifteen patients died of tumor progression during the follow-up, of whom 9 were at an advanced stage (Stage C) by the time of diagnosis and 6 at an early stage (Stage A/B). Kadish stage C patients had a significantly poorer outcome compared to the stage A/B patients (2-year survival rate: 71 versus 25%, resp.; *P* = .008) (Figure 2). Histopathologically high-grade patients (Grade 3/4) had a significantly poorer outcome when compared to the lower grade (Grade 1/2) patients (2-year survival rate: 16 versus 50%, resp.; *P* = .001) (Figure 3). Primary tumor extensions to orbital area had a significantly poorer outcome compared to the tumors with no extension to orbital area (2-year survival rate: 22 versus 60%, resp.; *P* = .042) (Figure 4). There was no statistically significant difference in survival rate between male and female patients (2-year survival rate: 40 versus 44%, resp.; *P* = .068). Patients with age <45 years had no poorer outcome compared to patients with age >45 years (2-year survival rate: 41 versus 43%; *P* = .059). Brain and bone marrow involvements showed poorer outcome compared to those without such involvement (Median survival rate: 9.1 versus 28.8 months, resp.; *P* = .001). Survival rate was not significantly different among pateints of Kadish C when stratified according to initial treatment (*P* = .8). 

### 4.4. Locoregional Recurrence and Distant Metastases

Twelve cases of recurrence occurred during follow-up periods (63%). Locoregional recurrence developed in 6 and distant metastasis in 6 patients. During the follow-up period, these groups exhibited distant metastasis to the brain, spinal cord, bone, bone marrow, meninges, and lungs. (Table 2). The median recurrence-free survival was 8.4 months (range, 1–124). As a treatment for recurrence and metastasis, reoperation of primary site plus radiation therapy in 4 patients and radiation therapy alone in 4 patients were offered. Combined radiation therapy and CTX were performed in 6 patients. The remaining 2 patients were treated with palliative care. Of the 12 cases of recurrence and metastasis, 10 patients died. Two patients were still alive at the time of preparation of this manuscript. 

## 5. Discussion

ONB is a rare malignant tumor that comprises 4,65% of malignant nasal and paranasal tumors [4]. Some 1000 cases have been reported in the English literature since it was first introduced in 1924 [5]. The incidence rate is bimodal, with peaks in the second and third decades of life and in the sixth and seventh decades of life and equal among females and males. As for our study, the age at diagnosis was most common in the fourth decade and female-to-male ratio was equal. The average time between the appearance of the first symptom and the diagnosis was reported as 6 months and this period was similar also in our group [6]. The most common symptoms at the initial diagnosis were defined as unilateral nasal obstruction and epistaxis in the literature. Our patients had similar symptoms at the initial diagnosis. There are no universally accepted diagnostic criteria for making the diagnosis of ONB. The characteristic histopathological morphology is a small blue cell neoplasm with a lobular architecture. This tumor may be misdiagnosed as an undifferentiated small cell carcinoma, melanoma, rhabdomyosarcoma, or other small blue cell neoplasms. Hyam proposed a grading system with grades 1–4 based on the presence or absence of seven different histopathological parameters. These are growth, architecture, mitotic activity, necrosis, nuclear pleomorphism, rosette formation, and fibrillary stroma [3]. 

Immunohistochemistry is a valuable diagnostic tool. Staining paterns for neuron-spesific enolase, chromogranin, synaptophysin, S-100, and epithelial markers can be helpful [7]. 

There is evidence that histological grade of ONB influences biologic behavior, particularly because it relates to disease progression, local recurrence, and metastasis. For the well-differentiated tumors a slower disease progression and less tendency for local recurrence have been described. In our study, the 2-year survival rate of this grade I/II-III/IV disease was 16 versus 50%, respectively. These observations suggest that the histopathological grading can have a prognostic value. More aggressive treatment, such as surgery, radiation, and intensive CTX, may be useful for patients with grade III/IV disease. A meta-analysis showed that the median overall survival at 5 years is 45% in 390 patients [8]. The distribution of ONB from 21 studies according to Kadish staging system was 12% at stage A, 27% at stage B, and 61% at stage C. The mean 5-year survival is 75% for stage A, 68% for stage B, and 41% for stage C [9]. The conclusions of the University of Virginia stated that Kadish stage is predictive of disease-related mortality [10]. However, in the series of the Mayo Clinic it was found that Kadish stage did not affect the outcome [11]. Danish clinicopathological study found that the staging system of Kadish was able to stratify into prognostically significant groups [11]. In our study, the 5-year survival rate of these tumors was 26% and median survival time was 23 months. The distribution of ONB according to Kadish staging system was 16% at stage A, 26% stage B, and 58% stage C. However, in our study, the 5-year survival rates of these stage groups were inferior compared to those reported in other studies. The cause of this poor result was presentation of 5 patients with distant metastases (26%; 2 brain, 1 bone marrow, 2 other sites), 4 patients (21%) with cervical lymph node metastasis, 12 patients (63%) with high-grade histology at the time of diagnosis, and inability to evaluate the surgical margins of the resected tumors. ONB can be disseminated both locally and distantly. The most common site of metastasis in published series is cervical lymph nodes. The survival rate of node positive patients is 29% compared with 64% of node negative patients [12]. Distant metastases have been described in the pancreas, the liver, mediastinum, bone marrow, lungs, leptomeninges, skin, and breasts [11, 12]. Cervical nodal metastases can develop in 17 to 33% of patients and distant metastases in 10 to 40% of patients over the course of disease [12, 13]. Craniofacial resection followed by RT seems to decrease recurrence to 10% [12]. Metastatic disease at presentation occurs in 10 to 50% of patients, depending on the study [6, 13, 14]. The most common site of metastatic spread is the cervical lymph nodes. Approximately 20 to 30% of metastatic disease involves the central nervous system [6]. Salvage therapy after local recurrence is possible in 30–35% of cases. In our group, there were 12 (63%) patients who relapsed after initial surgery. We found the local recurrence rate to be 31.5% and distant metastasis 31.5% after initial surgery. During the follow-up periods these groups exhibited distant metastases to the brain, spinal cord, bone, bone marrow, meninges, and lungs. Patients who had brain and bone marrow metastases had poorer prognosis than others having metastatic deposits of other sites. Involvement of the orbit is a serious prognostic factor, but when the orbital periosteum is affected without penetration into the orbital structures themselves, the eye may be preserved by resection and grafting of the periosteum without an adverse effect on survival as compared with orbital clearance. Irrespective to the most sophisticated imaging, involvement of the dura and orbital periosteum can accurately be determined only at surgery. In our study, involvement of the orbita is considered as a poor prognostic factor. This group's (8 patients) survival rate is poorer than those with no involvement of the orbita (*P* = .042). The Institute of Laryngology and Otology, University College London, published a study of 42 patients treated over 23 years. Craniofacial resection was used in all cases. Fifty-seven percent of patients received surgery combined with radiation therapy. Overall and disease-free survival rates were 77 and 61% at 5 years, and 53 and 42% at 10 years, respectively. Intracranial extension and orbital involvement were associated with worse outcome. Late recurrences were seen. Based on this experience, combined therapy with craniofacial resection and radiation therapy is recommended by this group [15]. In a retrospective study of 47 patients treated at multiple centers in Germany from 1979 to 2001, most of the patients were presented with stage C disease. The 5-year overall survival and event-free survival rate were 64% and 50%, respectively. Patients who received multimodality therapy had a significantly better event-free survival rate compared with those who did not receive multimodality therapy (74 versus 41%). The authors recommend a combination therapy including CTX, surgery, and postoperative radiation therapy for Kadish stage C patients [16]. In our study, initial treatment included surgery alone in three patients, RT with or without CTX in five, surgery plus postoperative RT in seven, and multimodality therapy (Surgery plus postoperative CTX plus postoperative RT) in three, only CTX in one patient. However, in our study choice of treatment modality did not show a statistically significant difference in survival rate. The reasons for that might be including low number of patients in the study, being 5 distant site metastasis, 4 cervical lymph node metastasis at the time of diagnosis, only one orbital exentration (although there were 8 orbital involvement), and 12 patients exhibiting high grade histology and inability to evaluate surgical margins in resected tumors.

In conclusion, advanced tumor stage, the initial histopathologic grade of III/IV, and involvement of orbita, brain, or bone marrow metastasis were the statistically significant poor prognostic factors, whereas age, sex, and treatment modality were not prognostic factors. Orbital exentration should be recommended to patients who have orbital involvement. Kadish C was a heterogeneous group. Only cervical metastasis was a better prognostic factor among other distant metastases. 

## Figures and Tables

**Table 1:** Tumor localization and symptoms at the time of diagnosis.

Patient no.	Tumor localization	Symptoms	First symptoms until time of diagnosis (months)
1	Sphenoid sinus-Nasal cavity	Rhinorrhea	3.7

2	Nasal cavity	Visual disturbance + Nasal obstruction + Rhinorrhea + Neck mass	1.8
3	Nasal cavity + Ethmoid + Maxilar sinus	Nasal obstruction	7

4	Nasal cavity	Nasal obstruction + Tears	9
5	Maxilar sinus	Nasal obstruction + Visual disturbance + Rhinorrhea + Neck mass	3.6

6	Sphenoid sinüs + Nasal cavite	Rhinorrhea	3.7
7	Ethmoid + Maxilar + Frontal + Orbita	Visual disturbance + Nasal obstrucsion + Rhinorrhea + Neck mass	1.8

8	Maxilar sinus + Orbita	Nasal obstruction + Epistaxis + Facial pain + Facial mass	25
9	Frontal sinus + Orbita	Eyeball pain	3

10	Maxilar sinus + Orbita	Teethache + Epistaxsis + Visual disturbance	9.2
11	Frontal sinus + Orbita	Exophthalmus	1.6

12	Nasal cavity	Nasal obstruction + Epistaxis + Facial pain + Facial mass	5.2
13	Ethmoid + Sphenoid sinus	Facial pain + Neck mass	29

14	Retropharyngeal	Facial paralysis + Earache	17.2
15	Nasal + Sphenoid + Orbita	Nasal obstruction + Exophthalmus + Epistaxis	8.1

16	Sphenoid sinus + Orbita	Headache + Proptosis	3
17	Sphenoid sinus + Orbita	Headache + Earache + Facial pain	4

18	Nasal cavity	Nasal obstruction + Epistaxis	24.5
19	Nasal cavity	Nasal obstruction + Epistaxis + Facial pain + Facial mass	6.5

**Table 2:** Patient characteristics, treatment modalities, and outcome.

Patient no.	Age	Sex	Grade	Kadish stage	Treatment	Reccurence	Time to reccurence (months)	Metastases	Treatment reccurence	Overall Survival (months)	Outcome
1	33	F	G 4	B	S + RT	Local	6.3	No distant metastasis	S + RT	67.8	DLD
2	40	M	G 4	C	RT + CTX	Distant site	5.2	Spinal cord + menengeal	RT	7.4	DMD
3	47	M	G 4	B	S + RT	Distant site	7.7	Brain	CTX + RT	9.3	DMD
4	30	F	G 2	A	RT + CTX	Local	70	No distant metastasis	CTX	90.1	Alive
5	63	M	Unk.	C	RT	Distant site	10.8	Lung	CTX	28.8	DMD
6	54	M	G2	A	S	Local	124	No distant metastasis	S + RT	152	DLD
7	17	M	G 4	C	S + RT	Distant site		Cervical lymph node + Menengeal + Bone marrow	RT	11.8	DMD
8	46	M	G 2	C	S + RT	Neck	23.6	Cervical lymph node	S + RT	99.4	DMD
9	67	F	Unk.	C	S + RT	Distant site		Brain + Bone marrow	RT + CTX	9.2	DMD
10	62	M	Unk.	C	RT	Local	7.3	No distant metastasis	RT + CTX	31	DLD
11	34	M	G 4	C	RT + CTX + S	Distant site	13.1	Brain	CTX + RT	9.7	DMD
12	33	M	G 3	B	RT + CTX + S	Local	12	No distant metastasis	RT	22.2	DLD
13	50	F	G 4	C	RT + CTX + S	Distant site	14.4	Spinal cord	CTX + RT	23.7	DMD
14	38	M	G 4	C	CTX	Distant site	8.5	Brain + Bone marrow	CTX + RT	20.7	DMD
15	45	M	G 4	C	S	Distant site		Brain	PC	3.4	DMD
16	40	F	G 4	C	S	Distant site		Brain	PC	3	DMD
17	68	F	G 3	B	S + RT	Distant site	66.5	Bone	RT	87	Alive
18	62	M	G 4	B	S	Local	11.4	No distant metastasis	S + RT	25.1	DLD
19	42	M	G 1	A	S + RT	No recurrence				72	Alive

CTX: chemotherapy; RT: radiotherapy; S: surgery; PC: palliative care; DLD: died of local disease; DMD: died of metastatic disease; G: grade; Unk: unknown.

**Table 3:** Treatment modalities.

Patient no.	Surgical procedure	CTX	Duration of CTX (Months)	Total radiotherapy fraction (Gy)
1	Craniofacial resection			50
2	—	cisplatin + etoposid	8	56
3	Craniofacial resection			65
4	—	cisplatin + etoposid ndoxan + vincristin + doxorubicin	12	65
5	—			60
6	Craniofacial resection			50
7	—			60
8	Maxillectomi + Orbital exenteration			50
9	Craniofacial resection			36
10	—			66
11	Craniofacia resection	cisplatin + etoposid	5	60
12	Craniofacial resection	cisplatin + etoposid	5	56
13	External thmoidectomy	cisplatin + etoposid cisplatin + vinkristin	10	60
14	—	cisplatin + etoposid carboplatin + etoposid	7	
15	Craniofacial resection			
16	Craniofacial resection			
17	Craniofacial resection	Cisplatin	1	70
18	Nasal resection			
19	Nasal resection			54

## References

[1] Bergen L, Luc G, Richard DL (1924). Esthesioneuroepithelioma olfactif. *Bulletin de l’Association Française pour l’Étude du Cancer*.

[2] Kadish S, Goodman M, Wang CC (1976). Olfactory neuroblastoma: a clinical analysis of 17 cases. *Cancer*.

[3] Hyams VJ, Hyams VJ, Baksakis JG, Michaels L (1998). Olfactory neuroblastoma. *Tumors of the Upper Respiratory Tract and Ear*.

[4] Spiro JD, Soo KC, Spiro RH (1995). Nonsquamous cell malignant neoplasms of the nasal cavities and paranasal sinuses. *Head and Neck*.

[5] Broich G, Pagliari A, Ottaviani F (1997). Esthesioneuroblastoma: a general review of the cases published sincethe discovery of the tumour in 1924. *Anticancer Research*.

[6] Dulguerov P, Calcaterra T (1992). Esthesioneuroblastoma: the UCLA experience 1970–1990. *Laryngoscope*.

[7] A-Oskouian RJ, Jane JA, Dumont AS (2002). Esthesioneuroblastoma: clinical presentation, radiological and pathological features, treatment, review of the literature, and the University of Virginia experience. *Neurosurg Focus*.

[8] Dulguerov P, Abdelkarim SA, Calcaterra TC (2001). Esthesioneuroblastoma: a meta-analysis and review. *The Lancet Oncology*.

[9] Elkon D, Hightower SI, Lim ML, Cantrell RW, Constable WC (1979). Esthesioneuroblastoma. *Cancer*.

[10] Polin RS, Sheehan JP, Chenelle AG (1998). The role of preoperative adjuvant treatment in the management of esthesioneuroblastoma: The University of Virginia experience. *Neurosurgery*.

[11] Foote RL, Morita A, Ebersold MJ (1993). Esthesioneuroblastoma: the role of adjuvant radiation therapy. *International Journal of Radiation Oncology Biology Physics*.

[12] Ingelholn P, Theilgaard SA, Buchwald C (2002). Esthesionneuroblastoma: a Danish clinicopathological study of 40 consecutive cases. *APMIS*.

[13] Bradley PJ, Jones NS, Robertson I (2003). Diagnosis and management of esthesioneuroblastoma. *Current Opinion in Otolaryngology & Head and Neck Surgery*.

[14] Chamberlain MC (2002). Treatment of intracranial metastatic esthesioneuroblastoma. *Cancer*.

[15] Lund AR, Howard D, Wei W, Spittle M (2003). Olfactory neuroblastoma:past, present, and future?. *Laryngoscope*.

[16] Eich HT, Hero B, Staar S (2003). Multimodality therapy including radiotherapy and chemotherapy improves event free survival in stage C esthesioneuroblastoma. *Strahlentherapie und Onkologie*.

